# From Gene Regulation to Gene Function: Regulatory Networks in *Bacillus Subtilis*

**DOI:** 10.1002/cfg.138

**Published:** 2002-02

**Authors:** Colin R. Harwood, Ivan Moszer

**Affiliations:** 1 Department of Microbiology and Immunology The Medical School University of Newcastle upon Tyne Framlington Place Newcastle upon Tyne NE2 4HH UK; 2 Unité de Génétique des Génomes Bactériens Institut Pasteur 28 rue du Docteur Roux 75724 Paris Cedex 15 France

## Abstract

*Bacillus subtilis* is a sporulating Gram-positive bacterium that lives primarily in the soil and associated water sources. The publication of the *B. subtilis* genome sequence and subsequent systematic functional analysis and gene regulation programmes, together with an extensive understanding of its biochemistry and physiology, makes this micro-organism a prime candidate in which to model regulatory networks *in silico.* In this paper we discuss combined molecular biological and bioinformatical approaches that are being developed to model this organism’s responses to changes in its environment.


**Comparative and Functional Genomics**
*Comp Funct Genom* 2002; **3**: 37–41.
**DOI:** 10.1002/cfg.138

**Conference Review**

### From gene regulation to gene function: regulatory networks in *Bacillus subtilis*

Colin R Harwood[1]* and Ivan Moszer[2]

[1] *Department of Microbiology and Immunology, University of Newcastle upon Tyne, Framlington Place, Newcastle upon Tyne, NE2 4HH, UK*
[2] *Unité de Génétique des Génomes Bactériens, Institut Pasteur, 28 rue du Docteur Roux, 75724 Paris Cedex 15, France*

**Correspondence to:*
*Department of Microbiology and Immunology, The Medical School, University of Newcastle upon Tyne, Framlington Place, Newcastle upon Tyne, NE2 4HH, UK.*
*E-mail: Colin.Harwood@ncl.ac.uk*

Received: 5 December 2001
Accepted: 6 December 2001
Published online:
20 December 2001

### Abstract

***Bacillus subtilis*** **is a sporulating Gram-positive bacterium that lives primarily in the soil and associated water sources. The publication of the *B. subtilis* genome sequence and subsequent systematic functional analysis and gene regulation programmes, together with an extensive understanding of its biochemistry and physiology, makes this micro-organism a prime candidate in which to model regulatory networks *in silico*. In this paper we discuss combined molecular biological and bioinformatical approaches that are being developed to model this organism's responses to changes in its environment. Copyright © 2001 John Wiley & Sons, Ltd.**

**Keywords: DNA arrays; functional analysis; gene expression; reporter genes; database conception; database integration**

### Introduction

*Bacillus subtilis* has been studied extensively over more than fifty years and is widely regarded as a model for the analysis of the medically, environmentally and industrially important Gram-positive bacteria. Knowledge of its biochemistry and physiology is extensive and *B. subtilis* strain 168 is highly amenable to genetic manipulation. *B. subtilis* and other *Bacillus* species are used in a wide range of industrial processes, for example for the production of extracellular enzymes, vitamins and fine biochemicals [12], and this industrial use has also enhanced our knowledge of the molecular and physiological characteristics of this bacterium.

### Post-genomic analyses

The genome sequence of *B. subtilis* was completed in 1997 [17] by a joint European/Japanese consortium. The genome is 4.2 Mbp in length and encodes approximately 4100 proteins. The completion of the genome sequence was followed by systematic programmes to elucidate the function of genes currently of unknown function and to study genome-wide gene expression. These collaborative programmes are designed to expand knowledge of the molecular biology of strain 168 and, ultimately, to construct a mechanistic model of its behaviour in laboratory-based studies. Data resulting from these programmes have been compiled into databases that are accessible over the Internet: SubtiList (**http://genolist.pasteur.fr/SubtiList/**), a dedicated DNA sequence database; Micado (**http://locus.jouy.inra.fr/cgi-bin/genmic/madbase/progs/madbase.operl/**), which has data on the characterisation of a collection of isogenic mutants; and Sub2D (**http://microbio2.biologie.uni-greifswald.de:8880/**), that holds data on the analysis of the *B. subtilis* 168 proteome. A new database, SubScript, for the storage and analysis of transcriptomic data, is currently under construction (see below).

Data from the genome and from the systematic programmes outlined above suggest that a significant proportion of the *B. subtilis* genome is dedicated to growth and survival in the extremely variable conditions found in the soil. Moreover, it has been proposed that a significant proportion of genes of unknown function (approximately 1800 or 40%) are required for survival. Thus, knowledge of the behaviour of *B. subtilis* in response to specific

Copyright © 2001 John Wiley & Sons, Ltd.

changes in its environment is likely to be of value for elucidating the role of genes of unknown function. To this end, proteomic and transcriptomic methodologies (eg. 2D-PAGE, DNA arrays, yeast two-hybrid analyses) are being directed towards defining regulons, and characterising their cognate regulatory proteins. A particularly valuable resource for the work is the existence of a collection of isogenic mutants (BFA mutants) in which the pMUTin integration vector has been introduced into virtually all genes on the *B. subtilis* chromosome [23]. pMUTin not only generates target gene mutants, but also links a *lacZ* transcriptional reporter to the target gene and a controllable promoter to any genes downstream and in the same operon. Interacting European (BACELL Network: **http://www.ncl.ac.uk/bacellnet/**) and Japanese (JAFAN: **http://bacillus.genome.ad.jp/BSORF-DB.html**) research consortia are using these mutants, in combination with post-genomic technologies such as DNA array and proteomics, to analyse *B. subtilis* gene function and regulatory networks in detail.

The response of *B. subtilis* to phosphate starvation illustrates the need to develop *in silico* techniques for modelling regulatory pathways. During phosphate starvation, *B. subtilis* responds by activating the phosphate stimulon, comprising, at least, the Pho and the $\sigma^B$-dependent general stress regulons [3,13,15,18]. Induction or repression of genes of the Pho regulon enables the cell to use limited phosphate resources more efficiently and to increase accessibility to alternative sources of phosphate. The Pho regulon is controlled by the interaction of at least 3 two-component signal transduction systems [15]. The centre of this regulatory network is the PhoP-PhoR sensor-regulator system [16]. Activation of the response regulator PhoP to PhoP$\sim$P during phosphate starvation leads to the induction or repression of genes in the Pho regulon. ResE, involved in the induction of genes during anaerobiosis, is required for the full induction of the Pho regulon, while a third response regulator, Spo0A, terminates the phosphate response, and initiates sporulation if phosphate starvation conditions persist. In addition to the phosphate starvation-specific Pho regulon, phosphate limitation also induces genes of the $\sigma^B$-dependent general stress ($\sigma^B$-GS) regulon [3]. The $\sigma^B$-GS regulon provides a non-specific resistance to stress, protecting DNA, membranes and proteins from the damage [1,11,13]. The activity of $\sigma^B$, an alternative sigma factor modulating promoter choice, is controlled by the anti-sigma factor RsbW. In unstressed cells, when the antagonist protein RsbV is phosphorylated (RsbV$\sim$P), RsbW can bind to and inactivate $\sigma^B$ [5]. In response to stress, RsbV is dephosphorylated [5,25,28], allowing it to sequestrate RsbW. As a result, $\sigma^B$ is free to activate genes in the $\sigma^B$-GS regulon [10]. Complex regulatory cascades control the phosphorylation state of RsbV [1,2,24,26,28].

Using a combination of reporter gene, DNA array and 2D-PAGE technologies, we have monitored genome-wide responses to phosphate starvation. *phoR*- and *sigB*-null mutants have been used to assign genes to the Pho and the $\sigma^B$-GS regulons, and to determine the effects of $\sigma^B$ on the expression of the Pho regulon and *vice versa*. The data indicated that the Pho and $\sigma^B$-GS regulons are interacting members of the phosphate stimulon (Prágai and Harwood, unpublished), although we do not, as yet, understand the molecular mechanisms underlying these interactions.

To date the analysis of the phosphate stimulon has been carried out by a hypothesis-driven approach. However, the extensive existing knowledge of this system, combined with high throughput data-rich transcriptomic and proteomic methodologies means that data-driven approaches can now be considered. To this end, we are attempting to use the signal transduction (including phosphorylation states and protein-protein interactions) and gene expression data to model the regulatory interactions within the phosphate starvation stimulon. The core of the current model is based around a gene expression weight matrix following the TreMM modelling strategy [27].

### Database integration

The wealth of information generated by large-scale genomic studies has to be collected and organized in specialised data structures. To this end, a number of dedicated databases have been constructed over the ten past years of *B. subtilis* genomics. Firstly the SubtiList database holds the core genomic data [20]. Developed in the framework of the genome sequencing project [17], SubtiList is the reference database for the *B. subtilis* 168 genome. It provides a curated dataset of DNA and protein sequences, combined with the relevant annotations and functional assignments. Information about gene functions and products is continuously updated by linking relevant

Copyright © 2001 John Wiley & Sons, Ltd. 

bibliographic references. Recently, sequence corrections arising from both systematic verifications and submissions by individual scientists were included in the reference genome sequence [19]. SubtiList is based on a generic relational data schema and World-Wide Web interface developed for the handling of bacterial genomes, called GenoList, which is used for a number of other organisms (**http://genolist.pasteur.fr/**). A user-friendly WWW interface was designed to allow users to browse easily through genome data and retrieve information according to common biological queries. SubtiList also provides more elaborate tools, such as pattern searching, which are tightly connected to the overall browsing system.

The second database, Micado [8,22], was developed for handling phenotypic data generated in the framework of the *B. subtilis* functional analysis programme [21]. Specific data structures and displays were developed both for the gel images used for authenticating the BFA mutant constructions, and for the graphs showing the growth and reporter gene activities of the mutants. Transcript and disruption maps are represented through generic physical maps, which are also used for navigating the complete genome. The Micado WWW interface was recently rewritten and its relational data model was ported on a new database management system. A comparable database is managed by JAFAN (**http://bacillus.genome.ad.jp/BSORF-DB.html**).

The third database dedicated to *B. subtilis*, Sub2D [6,7], was developed to analyse two-dimensional protein gels, with the aim of deciphering new regulons and stimulons. Sub2D was structured according to the guidelines established for the construction of two-dimensional protein databases [4]. It contains information about the methods of protein identification and the environmental conditions that influence the production of a given protein. Beside basic search forms and gene lists, proteins can be selected on the basis on their position on two-dimensional gels. Complex queries allow for the display of all the proteins similarly regulated by a given stimulus or controlled by a single transcriptional regulator.

More recently, two additional databases for *B. subtilis* genomics have been established in the framework of the current BACELL Network programme. Firstly, data generated by transcriptome analysis are organized into the SubScript database, which is still under active development. The objectives of SubScript are to provide both a data repository that can be browsed and queried *via* the Web, and a tool for the analysis of *B. subtilis* expression data, with the help of standard and original analysis methods. An elaborate data schema, comprising more than fifty classes, has been based on the recommendations published by the Microarray Gene Expression Databases (MGED) consortium (Minimal Information About Microarray Experiments, MIAME) [9], and the corresponding ArrayExpress database model (EBI: **http://www.ebi.ac.uk/arrayexpress/**). The original version of the schema has been superseded by a significantly improved version that is more appropriate to the precise requirements of the BACELL Network programme. This newer version was conceived to store experiment parameters and the raw data, as well as their subsequent analyses. A great deal of flexibility in the way experiments can be described has been anticipated in the data model. Specialised interfaces are currently being developed to allow users to enter, retrieve, and analyse expression data.

Finally, the newly developed SPID database contains information about two-hybrid protein interactions [14]. Graphic displays accessible through the WWW facilitate data mining and navigation through the interaction networks and the domains implied in protein pair interactions.

The five major databases on *B. subtilis* genomics described above generate an obvious requirement for better communication between their respective datasets. Indeed each of them is aimed to become a component of a comprehensive genomic resource for *B. subtilis*, gathering disparate classes of biological knowledge. This should allow investigators to pose complex questions across the various types of stored information. For example; which genes located close each other (SubtiList), are involved in sporulation (Micado), are co-activated in the presence of glucose in the medium (Sub2D and SubScript), and participate to a single protein complex (SPID)? Such enquiries require a level of data integration that has not currently been achieved, but which is under active investigation.

In the first instance, simple hypertext links through the WWW are being reinforced between the many *B. subtilis* databases. The use of invariant accession numbers greatly facilitates the definition of cross-references. This does not yet allow questions to be asked across the various data sets, but does reflect progress towards a greater degree of interaction between the databases that does not

Copyright © 2001 John Wiley & Sons, Ltd.

require challenging computing techniques: this approach is considered to be the most basic stage of database interoperation. At the opposite end of the data integration ladder is the data warehousing strategy. This approach implies that data schemas can be merged and that common software and technical implementation can be defined. The physical integration of heterogeneous datasets into one single repository, whilst probably the most efficient way of dealing with the complexities of elaborate queries, presents a number of drawbacks, including the need for regular data synchronisation. Universal data description and formalisation languages, such as XML, will help to reach this goal by facilitating exchange of structured data. As for the *B. subtilis* databases, a first level of data warehousing is being implemented naturally as two pairs of databases are being developed in the same locations (SubtiList/SubScript and Micado/SPID). Finally, as an intermediate solution, a truly dynamic database interoperability can be set up, using concepts such as those implemented in the CORBA protocols. This is an attractive solution as it allows the user to perceive one single and unified (virtual) data schema and to ask complex questions on it, while retaining the distributed data management. However, a number of limitations make this technology difficult to apply in practice, including implementation pitfalls and performance bottlenecks not present in the data warehousing approach (notably because of the network traffic). The CORBA architecture solution is being assessed within the small and recent SPID database.

Progress in defining shared knowledge representations, including the use of common nomenclatures, still has to occur in order to break some semantic barriers towards the definition of unified data models, allowing the use of any combination of selection criteria for retrieving information. The development of ontologies for some common and formal descriptions of biological concepts and entities may help to take this route.

### Acknowledgement

This work was supported by grants from the European Commission's Quality of Life programme (contract number QLG2-CT-1999-01455), the UK Biotechnology and Biological Sciences Research Council and the French Centre National de la Recherche Scientifique. Studies on the phosphate stimulon are being carried out by Zoltán Prágai and Nicola O'Conner, and the *in silico* modelling by Nicholas Allenby, Alan C. Ward and Anil Wipat. The development of the SubScript database is being performed by Sandrine Moreira.

### References

1. Akbar S, Kang CM, Gaidenko TA, Price CW. 1997. Modulator protein RsbR regulates environmental signalling in the general stress pathway of *Bacillus subtilis*. *Mol Microbiol* **24**: 567–578.
2. Akbar S, Gaidenko TA, Kang CM, O'Reilly M, Devine KM, Price CM. 2001. New family of regulators in the environmental signaling pathway which activates the general stress transcription factor $\sigma^B$ of *Bacillus subtilis*. *J Bacteriol* **183**: 1329–1338.
3. Antelmann H, Scharf C, Hecker M. 2000. Phosphate starvation-inducible proteins of *Bacillus subtilis*: proteomics and transcriptional analysis. *J Bacteriol* **182**: 4478–4490.
4. Appel RD, Bairoch A, Sanchez JC, *et al.* 1996. Federated two-dimensional electrophoresis database: a simple means of publishing two-dimensional electrophoresis data. *Electrophoresis* **17**: 540–546.
5. Benson AK, Haldenwang WG. 1993. *Bacillus subtilis* $\sigma^B$ is regulated by a binding protein (RsbW) that blocks its association with core RNA polymerase. *Proc Natl Acad Sci U S A* **90**: 2330–2334.
6. Bernhardt J, Büttner K, Scharf C, Hecker M. 1999. Dual channel imaging of two-dimensional electropherograms in *Bacillus subtilis*. *Electrophoresis* **20**: 2225–2240.
7. Bernhardt J, Büttner K, Coppée J-Y, *et al.* 2001. The contribution of the EC consortium to the two-dimensional protein index of *Bacillus subtilis*. In *Functional Analysis of Bacterial Genes: A Practical Manual*, Schumann W, Ehrlich SD, Ogasawara N (eds). John Wiley & Sons, Ltd: Chichester; 63–74.
8. Biaudet V, Samson F, Bessières P. 1997. Micado: a network-oriented database for microbial genomes. *Comput Appl Biosci* **13**: 431–438.
9. Brazma A, Hingamp P, Quackenbush J, *et al.* 2001. Minimum information about a microarray experiment (MIAME): toward standards for microarray data. *Nat Genet* **29**: 365–371.
10. Dufour A, Haldenwang WG. 1994. Interactions between a *Bacillus subtilis* anti-sigma factor (RsbW) and its antagonist (RsbV). *J Bacteriol* **176**: 1813–1820.
11. Gaidenko TA, Price CW. 1998. General stress transcription factor $\sigma^B$ and sporulation sigma factor $\sigma^H$ each contribute to survival of *Bacillus subtilis* under extreme conditions. *J Bacteriol* **180**: 3730–3733.
12. Harwood CR. 1992. *Bacillus subtilis* and its relatives: molecular biological and industrial workhorses. *Trends Biotech* **10**: 247–256.
13. Hecker M, Völker U. 1998. Non-specific, general and multiple stress resistance of growth-restricted *Bacillus subtilis* cells by the expression of the $\sigma^B$ regulon. *Mol Microbiol* **29**: 1129–1136.
14. Hoebeke M, Chiapello H, Noirot P, Bessières P. 2001. SPID: a *Bacillus subtilis* protein interaction database. *Bioinformatics* **17**: in press.

Copyright © 2001 John Wiley & Sons, Ltd. 

15. Hulett FM. 1996. The signal-transduction network for Pho regulation in *Bacillus subtilis*. *Mol Microbiol* **19**: 933–939.
16. Hulett FM, Lee J, Shi L, *et al*. 1994. Sequential action of two-component genetic switches regulates the PHO regulon in *Bacillus subtilis*. *J Bacteriol* **176**: 1348–1358.
17. Kunst F, Ogasawara N, Moszer I, *et al*. 1997. The complete genome sequence of the Gram-positive bacterium *Bacillus subtilis*. *Nature* **390**: 249–256.
18. Lahooti M, Harwood CR. 1999. Transcriptional analysis of the *Bacillus subtilis* teichuronic acid operon. *Microbiology* **145**: 3409–3417.
19. Médigue C, Rose M, Viari A, Danchin A. 1999. Detecting and analyzing DNA sequencing errors: toward a higher quality of the *Bacillus subtilis* genome sequence. *Genome Res* **9**: 1116–1127.
20. Moszer I, Jones LM, Moreira S, Fabry C, Danchin A. 2002. SubtiList: the reference database for the *Bacillus subtilis* genome. *Nucleic Acids Res* **30**: in press.
21. Ogasawara N. 2000. Systematic function analysis of *Bacillus subtilis* genes. *Res Microbiol* **151**: 129–134.
22. Samson F, Biaudet-Brunaud V, Gas S, *et al*. 2001. Micado, an integrative database dedicated to the functional analysis of *Bacillus subtilis* and microbial genomics. In *Functional Analysis of Bacterial Genes: A Practical Manual*, Schumann W, Ehrlich SD, Ogasawara N (eds). John Wiley & Sons, Ltd: Chichester; 45–52.
23. Vagner V, Dervyn E, Ehrlich SD. 1998. A vector for systematic gene inactivation in *Bacillus subtilis*. *Microbiology* **144**: 3097–3104.
24. Vijay K, Brody MS, Fredlund E, Price CW. 2000. A PP2C phosphatase containing a PAS domain is required to convey signals of energy stress to the $\sigma^B$ transcription factor of *Bacillus subtilis*. *Mol Microbiol* **35**: 180–185.
25. Völker U, Völker A, Haldenwang WG. 1996. Reactivation of the *Bacillus subtilis* anti-$\sigma^B$ antagonist, RsbV, by stress- or starvation-induced phosphatase activities. *J Bacteriol* **178**: 5456–5463.
26. Völker U, Luo T, Smirnova N, Haldenwang WG. 1997. Stress activation of *Bacillus subtilis* $\sigma^B$ can occur in the absence of the $\sigma^B$ negative regulator RsbX. *J Bacteriol* **179**: 1980–1984.
27. Weaver DC, Workman CT, Stormo GD. 1999. Modelling regulatory networks with weight matrices. *Pac Symp Biocomp* **4**:112–123.
28. Yang X, Kang CM, Brody MS, Price CW. 1996. Opposing pairs of serine protein kinases and phosphatases transmit signals of environmental stress to activate a bacterial transcription factor. *Genes Dev* **10**: 2265–2275.

Copyright © 2001 John Wiley & Sons, Ltd. 
